# The impact of the COVID-19 pandemic on enrollment in undergraduate health-related studies in Spain

**DOI:** 10.1186/s12909-023-04347-5

**Published:** 2023-05-26

**Authors:** Jaume-Miquel March-Amengual, Irene Cambra-Badii, Consolación Pineda Galán, Ester Busquets-Alibés, Montse Masó Aguado, Anna Ramon-Aribau, Lydia Feito Grande, Agustí Comella Cayuela, Nuria Terribas i Sala, Elena Andrade-Gómez, Naiara Martínez-Perez, Javier Jerez-Roig

**Affiliations:** 1 Research Group On Methodology, Methods, Models and Outcomes of Health and Social Sciences (M3O), Faculty of Health Sciences and Welfare, Center for Health and Social Care Research (CESS), University of Vic-Central University of Catalonia (UVic-UCC), Vic, Spain; 2 Chair in Medical Education, University of Vic-Central University of Catalonia (UVic-UCC), Vic, Spain; 3 Grífols Foundation Chair of Bioethics, University of Vic-Central University of Catalonia (UVic-UCC), Vic, Spain; 4 Department of Physiotherapy, University of Málaga, Málaga, Spain; 5 Faculty of Medicine, Complutense University of Madrid, Madrid, Spain; 6 Faculty of Health Sciences, La Rioja University, Logroño, Spain; 7 Department of Nursing, Faculty of Medicine and Nursing, University of the Basque Country UPV/EHU, Leioa, Spain

**Keywords:** COVID-19, Health, Education, Higher, Graduate, Students, Social change

## Abstract

The aim of this study was to determine whether the pandemic has reinforced the choice of pursuing health-related bachelor’s degrees, and to identify underlying factors that could contribute to that impact. This is a cross-sectional study using an online survey of 2,344 students of nursing, physiotherapy, medicine, psychology and podiatry who started health-related bachelor’s degrees after the COVID-19 outbreak in Spanish higher education institutions. The pandemic influenced the choice of these studies by increasing the desire to help others (33.2%), by increasing citizenship values (28.4%), and by increasing the desire to contribute to improving the situation of the country (27.5%). Women had a significantly greater influence on the increase in social values related to the practice of the profession produced by the pandemic, whereas men and the bachelor’s degree in podiatry were more influenced by salary prospects. An increased desire to help others was significantly higher among women and nursing and medical students. Podiatry and psychology were the degrees were most influenced by the pandemic, as more students decided to pursue them, something they had previously doubted, while in nursing, psychology, and medicine the pandemic reinforced their interest in pursuing the degree the most. Students personally affected by COVID-19 reported being more influenced in reconsidering their professional path and in reinforcing their desire to pursue the health-related studies.

**Supplementary Information:**

The online version contains supplementary material available at 10.1186/s12909-023-04347-5.

## Background

The SARS-CoV-2 outbreak and the subsequent COVID-19 pandemic started in December 2019 and was declared a global public health emergency in March 2020. Especially during 2020, it led to an overburdening of local healthcare systems and to lockdowns and other restrictive measures to control the spread of the disease. Frontline health professionals played a key role in the pandemic worldwide, facing uncertainty, excessive workloads, and high exposure to the virus [1, 2]. They also received social recognition and were called heroes, even though this term was seen as problematic [2].

One of the first focuses of research studies on the effects of the pandemic on college students was the impact on mental health [3–9]. New studies on the career path of health science undergraduate students also emerged, focusing on how career plans postgraduation were affected by the COVID-19 pandemic [6, 10–12]. In China, approximately one-fifth of medical students reported an increased inclination to become a clinician or to choose respiratory medicine and infectious diseases as their future specialties [10]. Several studies in the US on students of medicine [4, 10, 13], dentistry and dental hygiene [14], urology [15], and otolaryngology [16] showed that the COVID-19 pandemic may exert some influence on students’ future medical career choices.

A study carried out in China after the COVID-19 outbreak in 2020 reported that 6.7% of the students regretted their career choice because of bad experiences associated with identity, gender, and having experienced physical or verbal violence [17]. These changes were also studied particularly with dentistry students, which included concerns about employment, disruptions, uncertainty in pursuing alternative career plans, and long-term stability in the profession [14]. Studies conducted in the US and Canada identified that the pandemic affected the choice of medical specialty in approximately one-fifth of medical students [3, 4]. This may be attributed to significant barriers to progress in training [4, 5, 10, 18–20] and the negative impact of perceived mental stress during the COVID-19 pandemic [21].

On the other hand, studies on nursing students carried out in Japan [22] and in the US [23], and on medical students in China [24], showed that the pandemic reinforced the feeling of belonging to the profession and validated their interest. In China, 86% of nursing students pointed out a positive influence on the image of nursing [25]. Also in China, the reported choice of nursing as a future career increased from 51 to 63% after the COVID-19 pandemic [26]. In the US, nursing students reported that COVID-19 influenced their interest in this field [27].

Previous research on the impact of the COVID-19 pandemic on students’ careers has focused on their perceptions and ideas [4, 11, 20, 24, 28–30]. The current needs of society and the perception of a worldwide health crisis that extends in time may have increased interest in pursuing university degrees related to the health and social sectors. The constant messages in the media regarding the indispensable role of health professionals during the pandemic regarding prevention, diagnosis and treatment may have increased the interest and the interest and the degree of representation of health professions. Some previous research suggests that because of these messages students may have been highly motivated to pursue these bachelor’s degrees, both instrumentally and vocationally, meaning that their motivation to study was largely based on its perceived usefulness for their future success in their chosen profession [31, 32]. Motivation can be understood as a “major determinant of the quality of learning and success” [33], and it can be linked with several factors such as scientific interest in the health sciences, good job opportunities, willingness to help or serve others, family’s medical background and many more [34, 35].

To the best of our knowledge, no studies on the impact of the COVID-19 pandemic on the motivation of Spanish undergraduate students to pursue health sciences have been published. Therefore, we hypothesized that the COVID-19 pandemic increased students’ interest in choosing these degrees in Spain. We conducted this study with the aim of determining whether the pandemic has reinforced the choice of pursuing undergraduate degrees in health sciences, and to identify underlying factors that could contribute to that impact. The specific objectives are: 1) to describe motivational factors in choosing health-related bachelor’s degrees and to analyze differences by gender; 2) to analyze the impact of the COVID-19 pandemic in choosing a health-related bachelor’s degree in Spain by gender and degree; 3) to analyze the impact of the pandemic in choosing a health-related bachelor’s degree in Spain after being affected by COVID-19, i.e., students having contracted COVID-19 and students with severe COVID-19 or having relatives with severe COVID-19.

## Methods

### Design

This is an observational (cross-sectional) study using an online survey.

### Study population

The target population consisted of students (of any age and gender) who started, for the first time, health-related bachelor’s degrees (nursing, physiotherapy, medicine, psychology, occupational therapy, podiatry, pharmacy, nutrition, social work, biomedical engineering, biomedical sciences and human biology) after the COVID-19 pandemic – i.e., the academic years 2020–21 and 2021–22 – in Spanish higher education institutions (private, public or mixed).

We contacted higher education institutions in Spain to inform them about the project and ask for their participation. Then we used three strategies to reach students in the participating institutions: during a scheduled lecture (at least one project member joined the face-to-face or online lecture); through an e-mail or message with a link to the survey; and indirectly through social media (using Twitter and Instagram).

### Sampling selection and sample size

The sampling source was first-year undergraduate students enrolled in a Spanish university in nursing, physiotherapy, medicine, psychology, and podiatry bachelor’s degrees.

**Fig. 1:**
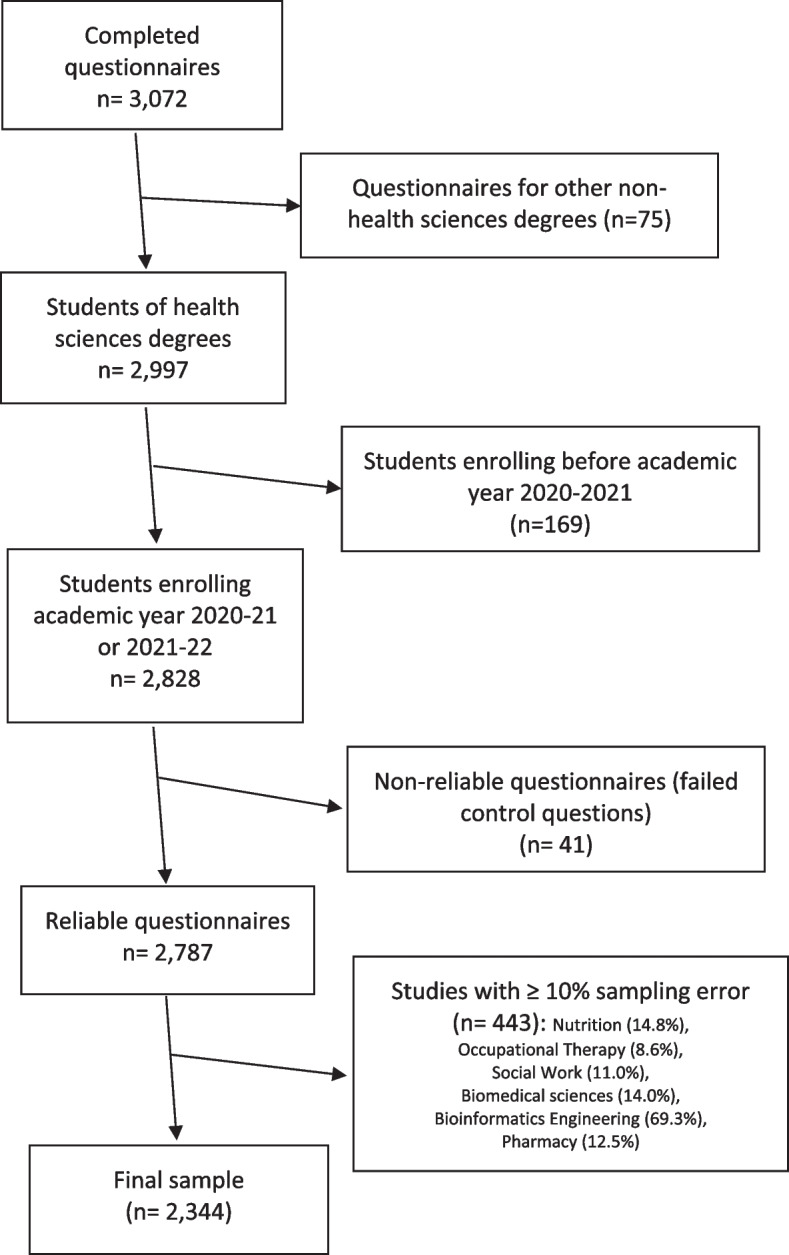
Sample selection flow-chart of health-related undergraduate students in Spain

- Inclusion criteria: students enrolled for the first time in the first year of these bachelor’s degrees in the academic years 2020–21 and 2021–22.
- Exclusion criteria: Students of other bachelor’s degrees, second-year students that have repeated a subject in the first year of the degree, students not enrolled in Spanish universities. To improve the veracity of the data, two control questions of the obvious correct answer type were included in the questionnaire: 1. The COVID-19 pandemic has made people wash their hands more frequently; 2. The COVID-19 pandemic has made people have to wear a mask in public places. Questionnaires in which the two control questions contained wrong answers (strongly or somewhat disagree) were excluded.
- Sample size: the study population included a total of 50,013 students divided into two cohorts, 25,001 students for the academic year 2020–21 and 25,012 for the academic year 2021–22. To calculate the representativeness of the sample, the number of places offered in Spanish universities for the academic years 2020–21 and 2021–22 was considered. This information is available in the database of the General Secretariat of Universities of the Ministry of Universities (Government of Spain) [36]. Only bachelor's degrees with a 10% or lower sampling error were included. Overall, 3,070 completed questionnaires from students from 34 Spanish universities were received. The response rate calculated from the number of accesses to the online questionnaire link (7,614) was 40.3%. After applying eligibility and exclusion criteria, 2,344 were included in this study (see Fig. 1): 844 nursing students, 602 physiotherapy students, 459 medical students, 308 psychology students and 131 podiatry students. 2,216 (94.5%) came from university recruitment and 128 (5.5%) came from recruitment via social networks. The rate of valid responses received was 30.8% (35.7% for responses received through university recruitment and 9.1% for recruitment through social networks).

The overall sampling error for both cohorts was 2.0% (95% CI) and 2.7% for cohort 20/21 and 3.0% for cohort 21/22 (see Supplementary Table 1).

### Data collection instrument and variables studied

The online survey was built with the *EncuestaFacil* platform (https://www.encuestafacil.com/) and consisted of sociodemographic information and data regarding the main factors influencing the students’ career choices and the impact of COVID-19 on their health, their social relationships and choice of degree. The self-reporting questionnaire was prepared by the research team based on previous studies [3, 22, 37].

The questionnaire was composed of 32 questions: 19 sociodemographic questions, 11 study questions on motivation and the impact of the pandemic on the choice of the bachelor’s degree, and 2 control questions. Most of the questions were closed-ended.

The following sociodemographic variables were included: age, gender, nationality, enrolled in a health-related bachelor’s degree, academic year (2020–21 or 2021–22), housing (family home, student housing, shared or individual place), study funding (family, work, scholarship and/or study loan), family monthly income (≤ 499, 500–999, 1000–1499, 1500–1999, 2000–2499, 2500–2999, 3000–4999, ≥ 5000 euros) and experience working in the socio-health and/or biomedical sector (no/yes). For the purpose of this study, we asked for students’ personal history of COVID-19 and COVID-19 cases (no/yes) among relatives, friends or cohabitants. If any response was affirmative, the participant was asked if the effects or progression of the illness were severe. The next set of questions/statements referred to eight possible factors affecting their choice of a health-related bachelor’s degree: willingness to help others, vocation, interest in the content, family tradition, job perspectives, ability to find a job, job prestige and good salary. Among these, students were asked to select up to three factors. The following set consisted of 10 questions/statements about the impact of the pandemic on: 1. reconsidering the career path; 2. studying the health-related degree (that was previously unclear); 3. further strengthening the willingness to pursue the health-related degree; 4. choosing the degree because of the desire to help others; 5. choosing the degree because of the desire to contribute to improving the situation in the country; 6. choosing the degree because it increased the feeling of belonging to the community; 7. choosing the degree because it increased citizenship values; 8. choosing the degree due to good employment prospects; 9. choosing the degree due to good salary prospects; 10. choosing the degree due to the increased prestige of the profession. A 5-point Likert scale from 1 (strongly disagree) to 5 (strongly agree) was created. Responses were dichotomized in 4 and 5 as high-very high impact versus 1–3 as no or low impact.

To test the questionnaire, a pilot study was conducted on 106 students from one Spanish university from March to April 2021. Participants were asked about the clarity of questions; only seven reported having problems understanding one or more questions, and their recommendations were considered for the final version of the survey. These modifications were not substantial, and these responses were included in the final sample.

### Administration procedure

The final survey was opened between 21 April 2021 and 24 May 2022.

- Methods for sending the survey: in most cases, the survey was sent by email, containing a brief presentation of the study with a link to the questionnaire to students enrolled in the participating universities. A brief face-to-face presentation explaining the purpose of the study and encouraging participation was also made at the partner universities. We introduced several control mechanisms associated with the online survey design. The Encuestafacil platform allows to configure a single and mandatory response to certain questions, which reduces response errors and blank data. In addition, several online mailboxes were used for data collection, one for each of the five partner universities of the project, one mailbox for other universities, and another mailbox for the collection of responses through recruitment via social networks.
- Incentives for participation: there were no incentives for participation.
- Response time: the estimated response time to the questionnaire was five minutes.
- Follow-up and reminders: several reminders encouraging participation were made at the participating universities.

### Data analysis

A descriptive analysis was performed to determine the characteristics of the sample. For quantitative variables, the mean and standard deviation were shown. None of the ordinal variables had normal distribution (Kolmogorov–Smirnov test). For the association of nominal variables, Pearson's Chi-square test was used. The Chi-square test for linear trend, also called linear by linear association, was used to analyze situations in which an ordered exposure variable contains three or more categories, and the outcome variable is of binary type. All analyses were performed with SPSS version 28.

Regarding the reliability of the questionnaire, it was calculated by Cronbach's alpha of the questionnaire, which was 0.873—exceeding the limit of 0.8 for basic research [38–40]. An exploratory factor analysis was performed with results of Kaiser–Meyer–Olkin (KMO 0.874 (*p* < 0.001). Total variance explained 60.1%, which is within the acceptable limit for social studies of 60% [41], with a factorial structure of 3 factors *(maximum likelihood extraction method and oblimin rotation factor)*. Factor 1 included social variables (Contributing to the country, Belonging to the community, Citizenship values, Helping others, Reinforcing willingness), factor 2 occupational variables (Salary prospects, Prospects for employment, Increased prestige), and factor 3 reconsideration variables (Previously unclear, Reconsidering professional path).

### Ethical aspects

The study was carried out in accordance with the Helsinki Declaration, the General Regulation (EU) 2016/679 of April 27 and the Spanish Organic Law 3/2018 of December 5 on the protection of personal data and guarantee of digital rights. Participation was voluntary and anonymous, and each participant was informed about the objectives of the project and the option to abandon the survey at any time. Informed consent was obtained from all the participants. The study was approved by the Ethics Committee of the University of Vic-Central University of Catalonia (code 145/2021).

## Results

Overall, 3,070 questionnaires from students of 34 Spanish universities were received. After applying eligibility criteria, 2,344 were included in this study (see Fig. 1): 844 nursing students, 602 physiotherapy students, 459 medical students, 308 psychology students and 131 podiatry students.

Table 1 includes a descriptive analysis of the sample. Most participants were female (1,767; 75.5%), and the mean age of the sample was 21.1; 2,025 (86.4%) were Spanish and 1,293 (55.2%) lived with their families; 1,786 (76.3%) students reported a family income between 1,000 and 4,999€. The majority of the students (1,891; 80.7%) had family support to pay for their studies, and 780 (33.3%) had a scholarship; 438 (19.2%) had work experience in the socio-health and/or biomedical sector, and of these 307 (70.3%) were active during the pandemic. Of the sample analyzed, 706 (30.1%) students reported having suffered from COVID-19, and 60 (2.6%) reported having serious complications or sequelae. 1,832 (78.2%) had a family member, cohabitant or close friend with COVID-19, and 483 (20.6%) had complications or severe effects.

**Table 1:** Sociodemographic characteristics of participants (*n* = 2,344)

Variables	Academic year 20–21	Academic year 21–22	Total
	***n* = 1,294**	***n* = 1,050**	***n* = 2,344**
	**n or mean (% or standard deviation)**		
**Gender**			
Female	1,006 (77.7)	761 (72.5)	1,767 (75.4)
Male	284 (21.9)	282 (26.9)	566 (24.1)
Other	4 (0.3)	7 (0.7)	11 (0.5)
**Age** (years), range 17–60	21.36 (6.26)	20.82 (5.82)	21.12 (0.07)
**Country of origin**			
Spain	1,118 (86.4)	907 (86.4)	2,025 (86.4)
Other from Europe	140 (10.8)	118 (11.2)	258 (11.0)
America	27 (2.1)	19 (1.8)	46 (2.0)
Africa	5 (0.4)	4 (0.4)	9 (0.4)
Asia	3 (0.2)	2 (0.2)	5 (0.2)
Oceania	1 (0.1)	0 (0)	1 (0.1)
**Regions**			
Andalusia	230 (17.8)	224 (21.3)	454 (19.4)
Aragon	38 (2.9)	18 (1.7)	56 (2.4)
Balearic Islands	40 (3.1)	54 (5.1)	94 (4.0)
Basque Country	126 (9.7)	105 (10.0)	231 (9.9)
Canary Islands	9 (0.7)	18 (1.7)	27 (1.2)
Cantabria	14 (1.1)	13 (1.2)	27 (1.2)
Castile and Leon	42 (3.2)	45 (4.3)	87 (3.7)
Castile-La Mancha	13 (1.0)	11 (1.0)	24 (1)
Catalonia	272 (21.0)	225 (21.4)	497 (21.2)
Community of Madrid	128 (9.9)	56 (5.3)	184 (7.8)
Extremadura	6 (0.5)	19 (1.8)	25 (1.1)
Galicia	97 (7.5)	24 (2.3)	121 (5.2)
La Rioja	15 (1.2)	12 (1.1)	27 (1.2)
Navarre	11 (0.9)	13 (1.2)	24 (1.0)
Principality of Asturias	11 (0.9)	5 (0.5)	16 (0.7)
Region of Murcia	11 (0.9)	7 (0.7)	18 (0.8)
Valencian Country	23 (1.8)	45 (4.3)	68 (2.9)
Foreigners	32 (2.5)	13 (1.2)	45 (1.9)
Not specified	176 (13.6)	143 (13.6)	319 (13.6)
**Bachelor**			
Nursing	520 (40.2)	324 (30.9)	844 (36.0)
Physiotherapy	339 (26.2)	263 (25.0)	602 (25.7)
Medicine	229 (17.7)	230 (21.9)	459 (19.6)
Psychology	167 (12.9)	141 (13.4)	308 (13.1)
Podiatry	39 (3.0)	92 (8.8)	131 (5.6)
**Housing**			
Family residence	798 (61.7)	495 (47.1)	1,293 (55.2)
Student residence	162 (12.5)	214 (20.4)	376 (16)
Shared place	275 (21.3)	275 (26.2)	550 (23.5)
Individual place	59 (4.6)	66 (6.3)	125 (5.3)
**Funding source**			
Family	1,037 (80.1)	854 (81.3)	1,891 (80.7)
Scholarship	443 (34.2)	337 (32.1)	780 (33.3)
Job	233 (18.0)	196 (18.7)	429 (18.3)
Loan	75 (5.8)	57 (5.4)	132 (5.6)
**Family income** (euros)			
≤ 499	86 (6.6)	80 (7.6)	166 (7.1)
500–999	130 (10)	83 (7.9)	213 (9.1)
1000–1499	244 (18.9)	161 (15.3)	405 (17.3)
1500–1999	191 (14.8)	148 (14.1)	339 (14.5)
2000–2499	191 (14.8)	156 (14.9)	347 (14.8)
2500–2999	154 (11.9)	131 (12.5)	285 (12.2)
3000–4999	215 (16.6)	195 (18.6)	410 (17.5)
≥ 5000	83 (6.4)	96 (9.1)	179 (7.6)
**Experience**^a^ (missing = 58)			
No	1,031 (83.1)	817 (78.1)	1,848 (80.8)
Yes	209 (16.9)	229 (21.9)	438 (19.2)
During the Pandemic	145 (69.4)	162 (71.1)	307 (70.3)

^a^Experience working in the socio-health and/or biomedical sector

Table 2 includes general factors affecting the choice of health-related bachelor's degrees. The most selected motivational factor was willingness to help others, followed by vocation and interest in the content. Family tradition, professional prestige and perspectives of a good salary, on the other hand, were the least selected items. When comparing genders, women were significantly more prone to choose a health-related bachelor's degree to help others (+ 12.3%) and vocation (+ 9.0%). Men were significantly more motivated by good salary prospects (+ 14.5%) and job prestige (+ 6.0%).

**Table 2:** Descriptive and bivariate analysis (by gender) of the motivational factors to choose a health-related degree (*n* = 2,333)

	**Total n (%)**	**Women n (%)**	**Men n (%)**	**Chi**	***p* value**
Helping others	1,812 (77.3)	1,419 (80.3)	385 (68)	36.896	< 0.001*
Vocation	1,381 (58.9)	1,079 (61.1)	295 (52.1)	14.165	< 0.001*
Interest in content	1,310 (55.9)	995 (56.3)	305 (53.9)	1.020	0.312
Good professional opportunities	749 (32)	542 (30.7)	203 (35.9)	5.317	0.021*
Good salary prospects	337 (14.4)	192 (10.9)	144 (25.4)	10.594	< 0.001*
Prospects for employment	298 (12.7)	218 (12.3)	79 (14)	1.013	0.314
Work prestige	199 (8.5)	125 (7.1)	74 (13.1)	19.780	< 0.001*
Family tradition	79 (3.4)	53 (3)	26 (4.6)	3.330	0.068

^*^Statistically significant (< 0.05)

Overall, 985 (42.0%) participants indicated that the pandemic reinforced their willingness to choose a health-related bachelor’s degree. In addition, a proportion of them reported that the pandemic had had an influence on the type of study they chose: 779 (33.2%) were motivated by the desire to help others, 666 (28.4%) by the desire to increase citizenship values, and 645 (27.5%) by the desire to improve the situation of the country. A lower proportion of students referred to a greater interest in choosing a bachelor’s degree for socioeconomic reasons: 594 (25.3%) because it increased employment prospects, and 497 (21.2%) because it increased professional prestige. The remaining factors/items had positive responses with a proportion of lower than 20.0%.

Table 3 shows the results of the bivariate analysis by gender. Women were significantly more influenced by the pandemic in wanting to study the career. They also had a significantly greater influence on the increase in social values related to the practice of the profession produced by the pandemic: reinforcement of the interest in choosing a health-related bachelor’s degree, helping others, citizenship values, contribution to the country, belonging to the community, reconsidering professional path and employment prospects. On the other hand, a significantly higher proportion of men reported choosing the degree for salary prospects.

**Table 3:** Analysis of the impact of the COVID-19 pandemic in choosing a health-related degree in Spain by gender (*n* = 2,333)

	**Total n (%)**	**[95% CI]**^**a**^	**Women n (%)**	**[95% CI]**^**a**^	**Men n (%)**	**[95% CI]**^**a**^	**Chi**	***p* value**
Reconsidering professional path	411 (17.5)	[0.16—0.191]	327 (18.5)	[0.167—0.204]	84 (14.8)	[0.12—0.18]	3.967	0.046*
Previously unclear	256 (10.9)	[0.097—0.123]	203 (11.5)	[0.1—0.131]	52 (9.2)	[0.069—0.119]	2.332	0.127
Reinforcing willingness	985 (42.0)	[0.4—0.441]	802 (45.4)	[0.43—0.477]	178 (31.4)	[0.276—0.355]	34.19	< 0.001*
Helping others	779 (33.2)	[0.313—0.352]	610 (34.5)	[0.323—0.368]	164 (29.0)	[0.253—0.329]	5.949	0.015*
Contributing to the country	645 (27.5)	[0.257—0.294]	529 (29.9)	[0.278—0.321]	113 (20.0)	[0.167—0.235]	21.377	< 0.001*
Belonging to the community	435 (18.6)	[0.17—0.202]	368 (20.8)	[0.19—0.228]	67 (11.8)	[0.093—0.148]	22.834	< 0.001*
Citizenship values	666 (28.4)	[0.266—0.303]	541 (30.6)	[0.285—0.328]	125 (22.1)	[0.187—0.257]	15.299	< 0.001*
Prospects for employment	594 (25.3)	[0.236—0.272]	469 (26.5)	[0.245—0.287]	124 (21.9)	[0.186—0.255]	4.856	0.028*
Salary prospects	374 (16.0)	[0.145—0.175]	260 (14.7)	[0.131—0.165]	112 (19.8)	[0.166—0.233]	8.234	0.004*
Increased prestige	497 (21.2)	[0.196—0.229]	369 (20.9)	[0.19—0.229]	127 (22.4)	[0.191—0.261]	.619	0.431

^*^Statistically significant

^a^One-sample binomial success rate (Clopper-Pearson)

In the analysis by bachelor’s degree (Table 4), there were significant differences in 5 of the 10 variables. More students of nursing (450; 53.3%), psychology (139; 45.1%) and medicine (200; 43.6%) reported that the pandemic reinforced their interest in choosing the degree. The influence of the pandemic on an increased desire to help others was significantly superior in nursing (319; 37.8%) and medicine (171; 37.3%). Also, the impact on the feeling of belonging to the community was stronger in students of nursing (190; 22.5%) and medicine (89; 19.4%). Podiatry was the degree with a significantly higher proportion of students (39; 29.8%) reporting the influence of the pandemic on salary prospects. Podiatry (136; 17.6%) and psychology (136; 16.9%) were the degrees in which more new students decided to enroll (before the pandemic they had different interests). Differences between degrees were not statistically significant for the remaining variables.

**Table 4:** Analysis of the impact of the COVID-19 pandemic in choosing a health-related degree in Spain, by bachelor (*n* = 2,344), according to linear-by-linear association test

	**Nursing n (%)**	**Physiotherapy n (%)**	**Medicine n (%)**	**Psychology n (%)**	**Podiatry n (%)**	**Linear-by-Linear Association**	***p* value**
Reconsidering professional path	175 (20.7)	75 (12.5)	55 (12.0)	83 (26.9)	23 (17.6)	45.365	0.913
Previously unclear	100 (11.8)	51 (8.5)	30 (6.5)	52 (16.9)	23 (17.6)	30.714	0.010*
Reinforcing willingness	450 (53.3)	158 (26.2)	200 (43.6)	139 (45.1)	38 (29.0)	116.478	< 0.001*
Helping others	319 (37.8)	169 (28.1)	171 (37.3)	91 (29.5)	29 (22.1)	27.646	0.001*
Contributing to the country	263 (31.2)	128 (21.3)	135 (29.4)	89 (28.9)	30 (27.5)	19.946	0.173
Belonging to the community	190 (22.5)	88 (14.6)	89 (19.4)	51 (16.6)	17 (13.0)	5.551	0.018*
Citizenship values	277 (32.8)	156 (25.9)	116 (25.3)	81 (26.3)	36 (27.5)	12.866	0.214
Prospects for employment	257 (30.5)	127 (21.1)	87 (19.0)	87 (28.2)	36 (27.5)	28.966	0.734
Salary prospects	136 (16.1)	106 (17.6)	55 (12.0)	38 (12.3)	39 (29.8)	28.296	0.001*
Increased prestige	196 (23.2)	113 (18.8)	84 (18.3)	77 (25.0)	27 (20.6)	9.192	0.778

^*^Statistically significant (< 0.05)

Table 5 shows the results of the bivariate analysis for the COVID-19 variables. Students infected by the disease presented a higher (+ 3.7%) influence of the pandemic on reconsidering their professional path. Students affected more, i.e., who themselves severely suffered the disease or who had relatives that did, reported being more influenced by the pandemic in the reconsideration of their professional path (+ 4.9%), in the reinforcement of their willingness to pursue a health-related bachelor’s degree (+ 5.6%) and in the recent decision to pursue it (+ 3.2%). The rest of the comparisons were not statistically significant.

**Table 5:** Analysis of the impact of the COVID-19 pandemic in choosing a health-related degree in Spain, after being affected by COVID-19 (*n* = 2,344), according to Chi square test

	**COVID-19**^**a**^				**Severe COVID-19**^**b**^			
	**Yes n (%)**	**No n (%)**	**Chi**	***p* value**	**Yes n (%)**	**No n (%)**	**Chi**	***p* value**
**Reconsidering professional path**	142 (20.1)	269 (16.4)	4.648	0.031*	115 (21.1)	296 (16.2)	9.54	0.002*
**Previously unclear**	81 (11.5)	175 (10.7)	0.316	0.574	70 (13.4)	186 (10.2)	4.35	0.037*
**Reinforcing willingness**	300 (42.5)	685 (41.8)	0.920	0.762	242 (46.4)	743 (40.8)	5.39	0.020*
**Helping others**	248 (31.1)	531 (32.4)	1.633	0.201	181 (34.7)	598 (32.8)	0.686	0.408
**Contributing to the country**	197 (27.9)	448 (27.4)	0.076	0.783	151 (29.0)	494 (27.1)	0.722	0.396
**Belonging to the community**	127 (18.0)	308 (18.8)	0.217	0.642	116 (22.3)	319 (17.5)	6.090	0.014*
**Citizenship values**	188 (26.6)	478 (29.2)	1.581	0.209	140 (26.9)	526 (28.9)	0.783	0.376
**Prospects for employment**	194 (27.5)	400 (24.4)	2.440	0.118	125 (24.0)	469 (25.7)	0.644	0.422
**Salary prospects**	127 (18.0)	247 (15.1)	3.114	0.078	75 (14.4)	299 (16.4)	1.216	0.270
**Increased prestige**	169 (23.9)	328 (20.0)	4.52	0.033*	107 (20.5)	390 (21.4)	0.178	0.673

^*^Statistically significant (< 0.05)

^a^Students infected by COVID-19

^b^Students with severe COVID-19 or having relatives with severe COVID-19

In order to better study the impact of the pandemic, students were initially asked from a set of factors which ones had made them study the degree and then asked about the influence the pandemic had had on these factors. Table 6 shows the students who were most influenced by the pandemic in their choice of degree. Students who initially did not indicate this factor as influencing their choice of degree and later stated that the pandemic had influenced them to consider this factor as a determinant determinant are the ones most influenced by the pandemic with regards to the factors studied.

**Table 6:** Descriptive and bivariate analysis (by gender) of the impact of the pandemic on the factors that most influenced students’ choice of degree. (*n* = 2,333)

	**Total**	**Women**	**Men**		
	**n (%)**	**n (%)**	**n (%)**	**Chi**	***p* value**
**Helping others**	106 (4.5)	74 (4.2)	31 (5.5)	1.658	0.198
**Prospects for employment**	473 (20.3)	376 (21.3)	97 (17.1)	4.548	0.033^*^
**Salary prospects**	253 (10.8)	193 (10.9)	60 (10.6)	0.046	0.830
**Increased prestige**	406 (17.4)	313 (17.7)	93 (16.4)	0.491	0.484

^*^Statistically significant (< 0.05)

Four hundred seventy-three students (20.3%) were influenced by the pandemic to study the degree because of good employment prospects. the occupation perspectives. This is the only factor that presents significant differences between men and women, where women have a higher percentage than men (+ 4.2%). Four hundred six students (17.4%) were influenced by the pandemic to choose the degree to increase their professional prestige, 253 (10.8%) because of good salary prospects and 106 (4.5%) because of the desire to help others.

## Discussion

The present study investigated the impact of the COVID-19 pandemic on the choice of health-related undergraduate studies in Spain, and one of its main findings was that the pandemic affected career choice and motivations in a significant proportion of students. This was more evident among students affected by COVID-19, since whether they themselves or a close relative suffered the disease; the pandemic reinforced their interest in pursuing a health-related bachelor's degree. Differences between genders and degrees were also observed for some determining factors.

Regarding the general motivating factors to pursue these types of studies, the most selected ones in our sample were the willingness to help others, vocation and interest in the content. Healthcare students usually pursue their future profession due to intrinsic factors such as personal motivations (e.g., interest in the sciences) and humanitarian factors (e.g., serve the community, help and care for others), sociodemographic factors (e.g. gender, socioeconomic status), extrinsic factors (e.g., employability, financial security, expectations of salary, diversity of job opportunities) and interpersonal factors (e.g., family, friends, prestige, social influence and recognition [34, 37]. According to a previous study, these mediators were altered during the COVID-19 pandemic, not only in the enrolled undergraduate students [14] but also in future students considering studying a health-related degree, because the main interests were focused on the desire to help others, reinforce citizenship values, and improve the country’s situation. The specific factors that can mediate the impact of the pandemic on undergraduate students may be classified in sociodemographic (e.g., age, gender), contextual and socioeconomic (income, urban area), health-related (e.g., mental health, overall health status) and pandemic-related (e.g., exposure to negative information, experiences during the pandemic) terms.

Most previous studies on the impact of COVID-19 on university students have focused on the effects on mental health due to the restrictions associated with the pandemic [42]. This phenomenon could have enhanced reflection on future plans of potential students wishing to study health-related higher education. Deng et al. [10] found that COVID-19 affected career choice both positively and negatively. In our study, we observed a positive impact of the pandemic on a proportion of students ranging from 16 to 42% depending on the determining factor. The latter frequency was found for the variable related to the increased willingness to pursue the degree and is higher than in other studies published.

A cross-sectional study conducted in China identified that, of 1,837 medical students, 11.7% were more willing and 6.9% less willing to become doctors after the COVID-19 outbreak. When it specifically came to majoring in respiratory medicine and infectious diseases, the percentages rose to almost 12% and 10%, respectively. Another cross-sectional study conducted in more than 1,000 students in China identified a 12% increase in the choice of nursing as a future career after the onset of the COVID-19 pandemic [26]. Other surveys conducted in the US found approximately 11–20% of dental and medical students believing that the pandemic would affect their future plans [14], including choice of residency and specialty [4, 13].

Previous studies have linked this interest to students’ motivations, since the pandemic had reinforced their interest in becoming a physician by validating their perception of the vital role that medical doctors play in society [24]. Medical students from the University of Geneva (Switzerland) highlighted the importance of the healthcare profession to society and confirmed their career choice during the pandemic [20]. Nursing students from the United States reported that the pandemic had influenced their interest in nursing, although the desire to help others was prior to the pandemic [27]. In a similar vein, we found that the pandemic influenced students’ desire to study the degree in order to help others (33.4%), to increase citizenship values (28.4%), and to contribute to improving the situation of the country (27.5%).

In our study, these social values related to professional practice (e.g., helping others, citizenship values, contribution to the country, belonging to the community) were found to be significantly more influential among women than men. On the other hand, men reported being more motivated by good salary prospects and job prestige. In the same vein, a previous study conducted in Catalonia (Spain) also found male physiotherapy students to be more interested in employability and income prospects [37]. Beyond the COVID-19 pandemic scenario, in a previous study female nursing students scored higher in values focused on social or affective relationships, while male nursing students scored higher in stereotypical masculine values such as dominance and success [43].

However, our results did not coincide with some other previous studies beyond the COVID-19 pandemic context. In our research, helping others was preponderant. Gennissen et al. [44] found different professional orientations or motivations in medical students; the main finding was that self-development throughout life was highly valued. Dzhaneryan & Gvozdeva [45] pointed out that female psychology students stated that the desire to build a career related to working for themselves was self-achievement. Other studies [46, 47] highlighted the entrepreneurial career ambitions in psychology students, who hoped to establish successful businesses and create employment opportunities for other people –with self-development in first place too.

When comparing degrees, we found a stronger influence of the pandemic on an increased desire to help others in nursing and medicine students. In relation to altruism, a qualitative study conducted in Japan found most nursing students stating that the pandemic reinforced their sense of belonging and decision-making processes. Several participants reported that, as committed citizens, they had the mission to increase the quality of the public health system and to serve their country during the disaster, making a personal sacrifice in seeking general rather than personal interests [22]. In the same vein, a survey conducted in France identified a high commitment of medical students to helping to manage the pandemic, reinforcing in most cases their motivation to become health professionals, even at a significant psychological cost [48]. Another study conducted with Chinese nursing students identified a high level of professional identity during the COVID-19 crisis, as well as a positive influence on the public image of the nursing profession [25].

The enhanced social perception of the importance of the healthcare workforce may have influenced future students, who may have seen this phenomenon as an opportunity for future professional development. In this sense, media coverage and governmental efforts to recognize the work of healthcare workers have played an important role in making health-related professions more visible during the pandemic [25]. The image of health-related disciplines has been highlighted to the general population, especially in the case of frontline healthcare workers, even invoking the language of ‘heroism’ to praise them [2]. It can be hypothesized that these factors could have caused a higher interest for undertaking a health-related undergraduate degree in previously undecided students or reinforced the desire to study a health-related degree in others. In the same vein, a longitudinal study conducted in China found that the COVID-19 outbreak strengthened students’ beliefs and choices to become good doctors in two-thirds of medical students, compared to only 13% reporting a negative impact on their career choices [49].

In the inferential analysis by degrees, we also observed that more students of nursing, psychology and medicine reported that the pandemic reinforced their interest in choosing the degree. In the same vein, a longitudinal study identified medicine and nursing as the degrees with the highest increased demand after the pandemic outbreak in Spain [50]. Interestingly, podiatry was the degree with a significantly higher influence of the pandemic on salary prospects and with more new students wishing to study it. In this line, a qualitative study conducted in the UK found that podiatry students were interested in the profession for its flexibility and good job prospects, including opportunities for career progression, salary and success [51].

In several factors, we found a significantly higher influence of the pandemic on the choice of a health-related bachelor’s degree among students more personally affected by COVID-19. Students that were more affected by the disease, i.e., who contracted the disease severely themselves or who had relatives that did, reinforced more their desire to study the degree, reconsidered more the professional path and recent decision to pursue the degree. Previous studies on students’ emotional distress during the COVID-19 pandemic have showed that most health risks to self or loved ones due to COVID-19 were not uniquely associated with emotional distress. Shanahan et al. [52] pointed out that pre-pandemic distress, secondary consequences of the pandemic (e.g. lifestyle and economic disruptions), and pre-pandemic social stressors were more consistently associated with young adults' emotional distress than COVID-19-related health risk exposures.

Coping mechanisms used by university students during the pandemic have been explored in previous studies, finding that a proactive developing stress management habits and personal resilience helped through the challenges of studying in the University and also enduring the stress of the pandemic [53, 54].

Future studies can focus on this aspect. Future studies may link motivation to professional values, and they can also be longitudinal, analyzing the transformative effects of a university education, beyond the influence of the pandemic on the main subjects of study. They may also describe the influence of macrosocial events, e.g., a macroeconomic crisis, on students’ university trajectories.

As one of the limitations of the study, the data collection instrument has not been fully validated, and only a preliminary exploratory analysis has been performed, in which the reliability of the questionnaire was calculated. We also performed a factor analysis, the first results of which are within the accepted margins for basic studies. Future studies can validate this kind of questionnaire and also perform a multivariate analysis on each motivational variable.

One of the strengths of the study is its sample, which includes university students from all over Spain. Despite using an online survey, we carefully analyzed all the questionnaires, especially those from social media recruitments, and we excluded unreliable questionnaires and degrees with a high sampling error to ensure data quality. However, like any study on the COVID-19 pandemic, the context must be taken into account, and its results cannot be generalized to the world population. The larger proportion of women in the sample can be seen as a limitation; however, this does replicate the proportion of women and men studying health sciences in Spain.

In conclusion, willingness to help others, vocation and interest in the content were the factors that most determined the choice of a health-related bachelor’s degree in this sample of Spanish students. Differences by gender show that women were more prone to choose a health-related bachelor's degree to help others and due to vocation, whereas men were more motivated by good salary prospects and job prestige. Regarding the impact of the pandemic, factors for choosing a health-related bachelor were strengthened in a proportion of students ranging from 11 to 42%. The most influential factors were the strengthening of the willingness to choose the degree, the desire to help others, the increase of citizenship values and the contribution to improving the situation of the country. Women were significantly more influenced by the pandemic in wanting to study the career, in reinforcing the interest in choosing the degree, helping others, citizenship values, contributing to the country, belonging to the community, reconsidering the professional path and employment prospects. Podiatry and psychology were the degrees that attracted more previously undecided students. More students of nursing, psychology and medicine reported that the pandemic reinforced their interest in choosing the degree. The influence of the pandemic on an increased desire to help others was significantly higher in nursing and medicine, whereas the feeling of belonging to community was stronger in nursing students. Podiatry was the degree with the highest proportion of students reporting an influence of the pandemic on choosing the degree due to good salary prospects. Students personally affected by the disease reported being more influenced in reconsidering their professional path, in reinforcing their willingness to pursue the health-related degree and in recently deciding to pursue it.

## Supplementary Information

**Additional file 1: Supplementary Table 1.** Sampling error.

**Additional file 2: Supplementary Table 2.** Questionnaire, questions and descriptives results (*n* = 2,344). Possible answers: 1 (strongly disagree) - 5 (strongly agree).

## Data Availability

The datasets used and/or analysed during the current study available from the corresponding author on reasonable request.
