# Supplementary material for: The impact of the COVID-19 pandemic on enrollment in undergraduate health-related studies in Spain
Source: BMC Med Educ. 2023 May 26;23:386. doi: 10.1186/s12909-023-04347-5 (PMC10214337; doi:10.1186/s12909-023-04347-5)
Supplement: Supplementary file 1 — Additional file 1: Supplementary Table 1. Sampling error. [file 12909_2023_4347_MOESM1_ESM.docx]

*Supplementary Table 1. Sampling error.*

|  | **Total population (Cohorts 1-2)** | **Total valid questionnaires** | | **Sampling error SE (CI 95%)** |
| --- | --- | --- | --- | --- |
| **Nursing** | 19,017 | 844 | 3.3% | |
| **Physiotherapy** | 6,405 | 602 | 3.8% | |
| **Medicine** | 11,465 | 459 | 4.5% | |
| **Psychology** | 11,871 | 308 | 5.5% | |
| **Podiatry** | 1,255 | 131 | 8.1% | |
| **TOTAL** | **50,013** | **2,344** | **2.0%** | |
