# Supplementary material for: The impact of the COVID-19 pandemic on enrollment in undergraduate health-related studies in Spain
Source: BMC Med Educ. 2023 May 26;23:386. doi: 10.1186/s12909-023-04347-5 (PMC10214337; doi:10.1186/s12909-023-04347-5)
Supplement: Supplementary file 2 — Additional file 2: Supplementary Table 2. Questionnaire, questions and descriptives results (n = 2,344). Possible answers: 1 (strongly disagree) - 5 (strongly agree). [file 12909_2023_4347_MOESM2_ESM.docx]

*Supplementary Table 2 Questionnaire, questions and descriptives results (n=2,344). Possible answers: 1 (strongly disagree) - 5 (strongly agree).*

| **Variable** | **Questionnaire questions in English / original Spanish** | **Average (SD) [95% CI]** |
| --- | --- | --- |
| **Reconsidering professional path** | The COVID-19 pandemic led me to reconsider my professional path. / La pandemia por COVID-19 me ha llevado a reconsiderar mi rumbo profesional. | 2.09 (1.333) [2.04 – 2.14] |
| **Previously unclear** | The COVID-19 pandemic led me to study this university degree, which I was previously unsure about. (La pandemia por COVID-19 me ha llevado a estudiar este grado universitario, cosa que antes dudaba). | 1.71 (1.156) [1.67 – 1.76] |
| **Reinforcing willingness** | The COVID-19 pandemic further strengthened my desire to pursue the degree I am studying. (La pandemia por COVID-19 ha fortalecido más mi voluntad de estudiar el grado que estoy cursando). | 3.05 (4.435) [3.00 – 3.11] |
| **Helping others** | The COVID-19 pandemic influenced me when choosing the degree due to my desire to help others. (La pandemia por COVID-19 me ha influido a la hora de elegir el grado por la voluntad de ayudar a los demás). | 2.73 (1.444) [2.67 – 2.78] |
| **Contributing to the country** | The COVID-19 pandemic influenced me when choosing the degree due to my desire to contribute to improving the situation of the country. (La pandemia por COVID-19 me ha influido a la hora de elegir el grado por la voluntad de contribuir a mejorar la situación del país). | 2.61 (1.369) [2.55 – 2.66] |
| **Belonging to the community** | The COVID-19 pandemic influenced me when choosing the degree because it increased my feeling of belonging to the community. (La pandemia por COVID-19 me ha influido a la hora de elegir el grado porque ha aumentado mi sentimiento de pertenencia a la comunidad). | 2.30 (1.289) [2.25 – 2.35] |
| **Citizenship values** | The COVID-19 pandemic influenced me when choosing the degree because it increased my civic values (La pandemia por COVID-19 me ha influido a la hora de elegir el grado porque ha aumentado mis valores ciudadanos). | 2.63 (1.352) [2.57 – 2.68] |
| **Prospects for employment** | The COVID-19 pandemic influenced me when choosing the degree due to the good employment prospects. (La pandemia por COVID-19 me ha influido a la hora de elegir el grado por las buenas perspectivas futuras de ocupación en la profesión). | 2.49 (1.342) [2.43 – 2.54] |
| **Salary prospects** | The COVID-19 pandemic influenced me when choosing the degree due to the good salary prospects. (La pandemia por COVID-19 me ha influido a la hora de elegir el grado por las buenas perspectivas de salario). | 2.13 (1.247) [2.07 – 2.18] |
| **Increased prestige** | The COVID-19 pandemic influenced me when choosing the degree that I am studying due to the increase in the prestige of the profession. (La pandemia por COVID-19 me ha influido a la hora de elegir el grado universitario que estoy cursando por el aumento del prestigio de la profesión). | 2.31 (1.311) [2.25 – 2.36] |
